# [^18^F]2-Fluoro-2-deoxy-D-glucose incorporation by AGS gastric adenocarcinoma cells *in vitro* during response to epirubicin, cisplatin and 5-fluorouracil

**DOI:** 10.1038/sj.bjc.6603971

**Published:** 2007-09-11

**Authors:** S A Suttie, K G M Park, T A D Smith

**Affiliations:** 1 Department of Surgery, School of Medical Sciences, University of Aberdeen, Foresterhill, Aberdeen AB25 2ZN, UK; 2 John Mallard PET Centre, Department of Biomedical Physics, School of Medical Sciences, University of Aberdeen, Foresterhill, Aberdeen AB25 2ZN, UK

**Keywords:** glucose transport, hexokinase, FDG-PET, apoptosis, gastric

## Abstract

Decreased tumour [^18^F]2-fluoro-2-deoxy-D-glucose (^18^FDG) incorporation is related to response however its significance at the cell level in gastro-oesophageal cancer and how it relates to cell death is unknown. Here human gastric adenocarcinoma (AGS) cells were treated with lethal dose 10 and 50 (LD_10_ and LD_50_), determined by using the MTT assay, of the three drugs, epirubicin, 5-fluorouracil and cisplatin, commonly used in the treatment of patients with gastro-oesophageal cancer. ^18^FDG incorporation was determined after 48 and 72 h of treatment with each drug and related to drug-induced changes in glucose transport, hexokinase activity, cell cycle distribution and annexin V-PE binding (a measure of apoptosis). Treatment of cells for 48 and 72 h with LD_50_ doses of cisplatin resulted in reductions in ^18^FDG incorporation of 27 and 25% respectively and of 5-fluorouracil reduced ^18^FDG incorporation by 34 and 33% respectively: epirubicin treatment reduced incorporation by 30 and 69% respectively. Cells that had been treated for 72 h with each drug were incubated in drug-free media for a further 6 days to determine their ability to recover. Comparison of the ability to recover from the chemotherapy agent, with ^18^FDG incorporation before the recovery period allowed an assessment of the predictive ability of ^18^FDG incorporation. Cells treated with either 5-fluorouracil or cisplatin demonstrated recovery on removal of the drug. In contrast, cells treated with epirubicin did not recover corresponding with the greatest 72 h treatment decrease in ^18^FDG incorporation. In contrast to adherent cells treated with cisplatin or 5-fluorouracil, adherent epirubicin-treated cells also exhibited very high levels of apoptosis. Glucose transport was decreased after each treatment whilst hexokinase activity was only decreased after 72 h of treatment with each drug. There was no consistent relationship observed between ^18^FDG incorporation and cell cycle distribution. Our results show that at the tumour cell level in gastric tumour cells, decreased ^18^FDG incorporation and glucose transport, accompanies therapeutic growth inhibition. ^18^FDG incorporation is particularly diminished in cells exhibiting apoptosis.

Combination chemotherapy may improve the length and quality of survival in a proportion of patients with advanced gastro-oesophageal cancer. With currently available chemotherapy regimens, only 40–60% of patients will respond to treatment (Gilbert *et al*, 2002). Present chemotherapy regimens for gastro-oesophageal cancer include epirubicin, cisplatin and 5-fluorouracil (5-FU). If patients are to avoid unnecessary treatment with particularly toxic agents, it is important that non-responders are identified at an early stage.

A number of studies (Couper *et al*, 1998; Weber *et al*, 2001; Kroep *et al*, 2003; Ott *et al*, 2003) have demonstrated that a reduction in tumour uptake of the glucose analogue, [^18^F]2-fluoro-2-deoxy-D-glucose (^18^FDG), is observed during and upon completion of chemotherapy in gastro-oesophageal cancer. Although decreased tumour ^18^FDG uptake has been shown to be associated with response (and may in fact predict tumour response), its significance at the gastro-oesophageal tumour cell level is not known. Response to chemotherapy, as seen on ^18^FDG-PET with a corresponding reduction in uptake in solid tumours, may be related to a decrease in tumour cellularity or a decrease in ^18^FDG uptake per cell, or a combination of both. In solid tumours, tumour cells can recover and it is not known if this decreased ^18^FDG uptake during response is due to modulation in ^18^FDG uptake by cells that will not recover. This reduction in cellular uptake of ^18^FDG may occur prior to reduction in tumour volume and therefore may reflect apoptosis. To further confuse matters, a flare phenomenon has been recorded in the early period following exposure to chemotherapy (Basu and Alavi, 2007). In addition chemotherapy may alter ^18^FDG cellular uptake by hexokinase (HK) and/or glucose transport modulation.

The fluorinated glucose analogue, ^18^FDG, is transported into tumour cells via a family of glucose transporter proteins, then phosphorylated by the enzyme HK to ^18^FDG-6-phosphate after which it undergoes little further metabolism. Due to low levels of G-6-Pase in tumour cells this is considered an irreversible reaction (Weber and Cantero, 1955; Warburg, 1956; Gallagher *et al*, 1978). Higher levels of HK, namely, HK2 (Torizuka *et al*, 1995; Mathupala *et al*, 1997) and GLUT, especially GLUT1 (Mueckler, 1994; Younes *et al*, 1996; Smith, 1999) and low levels of G-6-Pase (Nelson *et al*, 1996) have been reported in tumour tissue compared with corresponding normal tissue. Both glut proteins and HK activity have been implicated as the rate limiting step in cellular ^18^FDG uptake (Higashi *et al*, 1997; Waki *et al*, 1998; Brown *et al*, 1999; Kurokawa *et al*, 2004; Tohma *et al*, 2005; Zhao *et al*, 2005), although plasma (or media) glucose concentrations may have more of an impact (Aloj *et al*, 1999; Zhao *et al*, 2002; Burrows *et al*, 2004).

We have determined the effect of three commonly used chemotherapeutic agents (epirubicin, cisplatin and 5-FU) in the treatment of upper gastro-intestinal tumours on the cellular incorporation of ^18^FDG and on steps associated with its incorporation, that is, glucose transport and HK activity. As tumour cells within solid tumours are likely to be exposed to different drug concentrations, we have treated cells with both lethal dose 50 (LD_50_) and drug doses that have a low cell growth inhibition (LD_10_, 5–10% cell death). Previous work on AGS (human gastric adenocarcinoma) cells has shown that they can recover from treatment with cytotoxic doses of combinational regimes of 5-FU and cisplatin but not when epirubicin is included in the combination (Couper and Park, 2003). However, the design of Coupers' study meant that this effect could not be specifically related to epirubicin rather than triple combination chemotherapy. Our study evaluated the effect of each individual chemotherapeutic agent. Growth curves for AGS cells were established after exposure to each epirubicin, cisplatin and 5-FU, with LD_10_ and LD_50_ established for each agent at 48 h. Growth curves were taken to 216 h to determine the extent to which cells were irreversibly damaged by the chemotherapeutic agents. All subsequent experiments on ^18^FDG uptake and its relationships to glucose transport, HK activity, cell viability, cell cycling and apoptosis were undertaken at 48 and 72 h.

The aims of this study were to elucidate the effect, on cellular (AGS cells) ^18^FDG incorporation, of exposure to three commonly used chemotherapeutic agents in the treatment of gastro-oesophageal cancer and to determine the mechanisms behind these changes in ^18^FDG cellular incorporation and their relationship with apoptosis.

## MATERIALS AND METHODS

### Cell line

A human gastric adenocarcinoma cell line (AGS) (ECACC, Porton Down, Salisbury, UK) was cultured in HAM F-12 media (Sigma, Dorset, UK) (supplemented with sodium hydrogen carbonate, penicillin G, streptomycin sulphate and L-glutamine and 10% fetal calf serum (Labtech International, East Sussex, UK)) at 37°C in a humidified incubator with 5% CO_2_ : 95% air.

The cells were subcultured in vented 80 cm^2^ flasks (Nunclon Delta Surface, Roskilde, Denmark). Cell suspensions were obtained by trypsinisation (ethylenediaminetetraacetic acid (EDTA)/trypsin) (Sigma) of the adherent cell monolayer, with 5 ml of EDTA/trypsin, when cells reached 70–80% confluence. Cell counts were performed using a haemocytometer (Bright-Line Haemocytometer, Sigma).

A seeding density of 7500 cells per 0.35 cm^2^, based on previous work (Couper and Park, 2003), was used for all experiments.

### Cytotoxicity assays

These were performed to identify the LD_10_ (5–10% cell death) and LD_50_ doses of three chemotherapeutic agents, commonly used in the treatment of gastro-oesophageal cancers, using the MTT (3-(4,5-dimethylthiazol-2-yl)-2,5-diphenyl tetrazolium bromide) assay (Mosmann, 1983; Carmichael *et al*, 1987).

Cells were plated in HAM F-12 media, at optimal seeding density, and returned to the incubator for 24 h to allow cell adherence to the plate. Media supplemented with varying concentrations of the chemotherapeutic agents epirubicin (Pharmacia and Upjohn Ltd., Milton Keynes, UK) (0.0039–0.5 *μ*g ml^−1^), cisplatin (Faulding Pharmaceuticals, Warwickshire, UK) (0.156–20 *μ*g ml^−1^) and 5-FU (Faulding Pharmaceuticals) (0.156–80 *μ*g ml^−1^) was then added and the plate returned to the incubator for either 48 or 72 h. A background of media and a control of media and cells were also set up.

After 48 or 72 h of exposure to the chemotherapy agents, MTT (Sigma) was added and then incubated for a further 4 h at 37°C. Using a scanning multi-well spectrophotometer (Dynatech MR5000, Dynatech Labarotaries Inc., Chantilly, VA, USA) measuring spectrophotometric absorbance at 570 nm, the plates were analysed using Biolinx 2.0 software (Biolinx 2.0, Dynatech Labarotaries Inc.). All experiments were repeated three times with six replicates per experiment. All subsequent cell work was based on the LD_10_ and LD_50_ concentrations of the chemotherapy agents at 48 h so that time was the only variable.

### Quantifying ^18^FDG uptake

Cells were seeded and incubated for 24 h, with control flasks were seeded at half the optimum seeding density so that cell density was similar in the control and treated flasks at the time of the ^18^FDG uptake measurement. Control, LD_10_ and LD_50_ flasks were set up in triplicate and the corresponding concentrations of each chemotherapeutic agent and returned to the incubator for either 48 or 72 h. The incorporation of ^18^FDG (obtained from the John Mallard PET Centre, Aberdeen) was determined by incubation of treated and control cells with ^18^FDG (1 kBq ml^−1^) for 20 min at 37°C followed by rapid washing with phosphate buffered saline (PBS) as described previously (Smith *et al*, 2006). ^18^FDG uptake was expressed relative to protein content (milligram of cellular protein) and per treated flask.

### Protein assay

Protein content was assessed by the bicinchoninic acid protein assay kit according to the manufacturers' instructions (Sigma).

### Glucose transport

Initially the linear phase of [^3^H]*O*-methylglucose (^3^H-OMG) uptake, a measure of glucose transport (Cloherty *et al*, 2002), was determined by incubating cells with ^3^H-OMG for 1, 2, 3, 5, 15 and 30 s. ^3^H-OMG uptake in control AGS cells was very rapid, with the linear part of the time activity curve for ^3^H-OMG uptake at 37°C complete within 2 s of beginning incubation. Experiments were, therefore, conducted at 25°C with an exposure to ^3^H-OMG of 1 s.

Glucose transport rates were determined at both 48 and 72 h following the addition of required dose of chemotherapeutic agent, by incubation with media and ^3^H-OMG (0.5 *μ*Ci ml^−1^ (specific activity 111GBq mmol^−1^)), at 25°C as described previously (Smith *et al*, 2006) except that the incubations were performed for 1 s. ^3^H-OMGuptake was expressed in terms of protein content (milligram of cellular protein).

### Hexokinase activity assay

AGS cells were set up and treated with chemotherapy as per ^18^FDG uptake. Following the incubation for 48 or 72 h with chemotherapy the media was removed, and each flask was washed twice with ice-cold PBS. Cells were then trypsinised and centrifuged with the cell pellet washed a further two times with ice-cold PBS. The resulting cell pellet was then stored at −70°C until the HK activity was assessed based on a modification to the methods by Miccoli *et al* (1996) as described previously (Smith *et al*, 2006).

Enzyme activity was expressed as mU mg^−1^ cellular protein using the extinction coefficient for NADPH of 6.3 × 10^3^ mol^−1^ cm^−1^.

### Flow cytometry DNA quantification

AGS cells were set up and incubated in chemotherapy as previously described for ^18^FDG uptake. After the required incubation period the media was discarded and the cells were harvested and cell cycle distribution was determined as described previously (Al-Saeedi *et al*, 2005).

### Cell regrowth following exposure to chemotherapy

Cells were plated as for MTT assay and treated for 72 h with LD_50_ and LD_10_ doses of each chemotherapy agent. Following this, the media was removed and the cells were washed with warm (37°C) PBS to remove any traces of residual chemotherapy. Fresh media (without chemotherapy) was then added and the plates were returned to the incubator. After 24, 48, 72, 96, 120 and 144 h incubation, MTT assays were performed to determine the latent cytotoxicity of the chemotherapeutic agents. Each experiment was repeated in triplicate with six replicates for each agent and time point.

### Annexin V-PE flow cytometry

Annexin V flow cytometry was used to discriminate between intact cells, early apoptotic and late apoptotic or necrotic cells.

AGS cells were set up as per ^18^FDG uptake. Control and cells treated for 72 h with chemotherapy agents were detached by incubating the cells in non-enzymatic cell dissociation solution in PBS (Sigma) and added to fresh media. The cell concentration was then adjusted to 3 × 10^5^ cells ml^−1^, and 1 ml of this cell suspension was then transferred to FACS tubes and centrifuged. The cell pellet was re-suspended in 1 ml of binding buffer (140 mM sodium chloride, 25 mM calcium chloride, 10 mM HEPES (*N*-(2-hydroxyethyl)piperazine-*N*′-(2-ethanesulphonic acid) hemisodium salt), 500 ml distilled water; Sigma). Following further centrifugation and removal of the supernatant, 15 *μ*l of annexin V staining buffer (10 *μ*l Via-Probe (7-N actinomycin D), 5 *μ*l annexin V-PE) (BD Biosciences Pharmingen, Oxford, UK) was added to each cell sample. The samples were stored in the dark at room temperature for 15 min after which 400 *μ*l of binding buffer was added and annexin V flow cytometry was performed within 1 h, analysing 10 000 cell events.

Annexin V flow cytometry was performed on a Becton Dickinson FACS Calibur (San Jose, CA, USA), and results analysed using CellQuest software (Becton Dickinson).

### Statistics

Results are expressed as means, ±s.e.m.. ^18^FDG, ^3^H-OMG uptake and HK activity were expressed as a percentage of the control. Significance of difference between means was determined by using the paired *t*-test (Statistical Package for the Social Sciences V13.1, SPSS Inc., Chicago, IL, USA), with a *P*-value of <0.05 indicating statistical significance.

## RESULTS

### Cytotoxicity assay

Cytotoxic doses resulting in a 5–10% decrease in cell number (LD_10_) and LD_50_ doses of chemotherapy after 48 h exposure were for cisplatin 0.156 and 5 *μ*g ml^−1^, 5-FU 0.156 and 20 *μ*g ml^−1^ and epirubicin 0.0039 and 0.125 *μ*g ml^−1^ respectively (Table 1).

### Effect of chemotherapy on ^18^FDG uptake

^18^FDG uptake per untreated control flask (expressed as cpm per 20 min per flask) was 8714±1182 and 14 753±986 respectively after 48 and 72 h incubation periods. ^18^FDG uptake, expressed as counts per milligram of cells per minute in untreated control flasks, was 783±94 and 984±35 (cpm per 20 min per mg protein) respectively following 48 and 72 h incubation periods.

**Figure 1:**
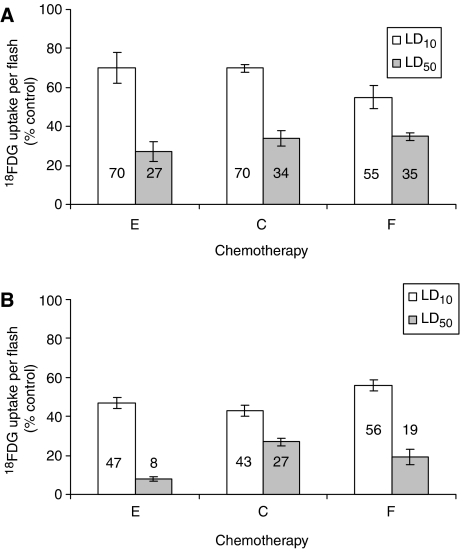
Cellular [^18^F]2-fluoro-2-deoxy-D-glucose (^18^FDG) incorporation, per treated flask, after 48 (**A**) and 72 (**B**) h treatment with lethal dose 10 (LD_10_) (white) and LD_50_ (grey) doses of epirubicin, 5-FU or cisplatin expressed as a percentage of incorporation by untreated controls (E=epirubicin, C=cisplatin, F=5-fluorouracil).

All three chemotherapeutic agents caused a reduction in ^18^FDG uptake per flask of treated cells (Figure 1A and B), with the greatest reduction occurring following 72 h exposure to epirubicin. Upon 48 h exposure to LD_10_ epirubicin, there was a non-significant reduction in ^18^FDG uptake per flask (*P*=0.104), with a significant reduction upon exposure to LD_50_ (*P*=0.006) compared with untreated controls. Both 48 h LD_10_ and LD_50_ exposure to cisplatin and 5-FU resulted in a significant reduction in ^18^FDG uptake per flask (cisplatin, *P*=0.037 and *P*=0.001; 5-FU, *P*=0.001 and *P*=0.001). Compared with untreated controls there was a significant reduction in ^18^FDG uptake per flask following 72 h LD_10_ and LD_50_ exposure to epirubicin (*P*=0.001 and *P*=0.001 respectively), cisplatin (*P*=0.001 and *P*=0.001 respectively) and 5-FU (*P*=0.001 and *P*=0.001 respectively).

**Figure 2:**
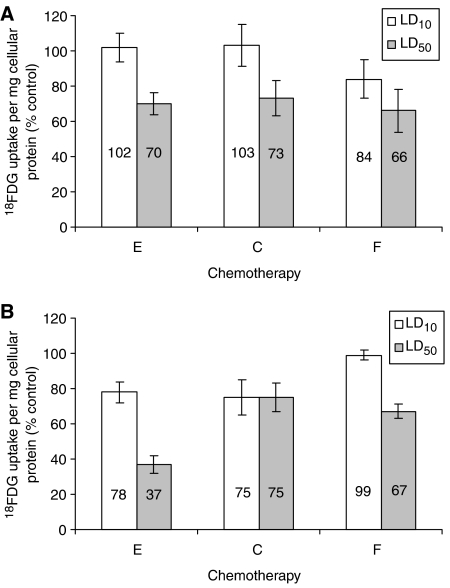
Cellular [^18^F]2-fluoro-2-deoxy-D-glucose (^18^FDG) incorporation, per milligram cellular protein, after 48 (**A**) and 72 (**B**) h treatment with lethal dose 10 (LD_10_) (white) and LD_50_ (grey) doses of epirubicin, 5-FU or cisplatin expressed as a percentage of incorporation by untreated controls (E=epirubicin, C=cisplatin, F=5-fluorouracil).

Exposure to all three chemotherapeutic agents resulted in a reduction in cellular ^18^FDG uptake, with the greatest reduction (63%) occurring on exposure to LD_50_ epirubicin after 72 h exposure (Figure 2A and B). This dose also produced a high level of cell death (69%). Compared with untreated controls there was a significant reduction in ^18^FDG uptake following 72 h LD_10_ (*P*=0.003) and both 48 and 72 h exposure to LD_50_ epirubicin (*P*=0.001 and *P*=0.011 respectively), with a non-significant reduction in ^18^FDG uptake following 48 h incubation at LD_10_ levels (*P*=0.789). ^18^FDG uptake was significantly decreased in cells exposed to LD_10_ (*P*=0.01) and LD_50_ (*P*=0.001) doses of 5-FU for 48 h exposure. Exposure to 5-FU for 72 h resulted in a significant decrease in ^18^FDG uptake at LD_50_ concentration (*P*=0.001) whilst there was little difference between control and LD_10_ (*P*=0.839). Exposure for 48 h to cisplatin, LD_10_ had little effect on ^18^FDG uptake (*P*=0.636) in contrast to exposure of LD_50_, which resulted in a significant reduction in tracer uptake (*P*=0.001). After 72 h exposure to cisplatin there was little further reduction in ^18^FDG cellular uptake compared with exposure to 48 h LD_50_.

### Effect of chemotherapy on cell cycle

Exposure to LD_50_ cisplatin and epirubicin resulted in cell cycle arrest in G_2_ phase, with LD_10_ doses resulting in G_1_ arrest at both 48 and 72 h incubation periods. LD_10_ dose of 5-FU resulted in S phase arrest, whilst the LD_50_ caused G_1_ arrest, irrespective of incubation periods (Table 2).

### Effect of chemotherapy on glucose transport

**Figure 3:**
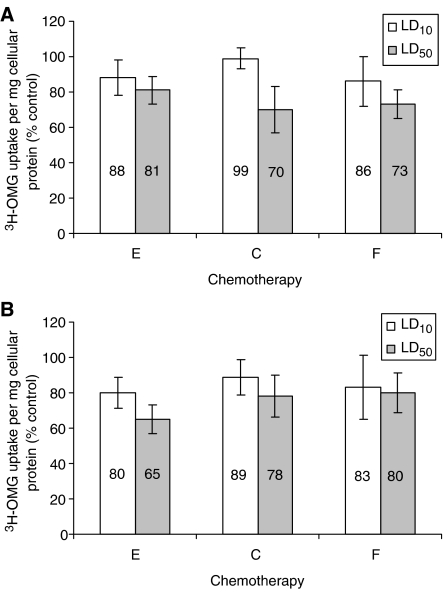
Cellular [^3^H]*O*-methylglucose (^3^H-OMG) uptake, per milligram of cellular protein, after 48 (**A**) and 72 (**B**) h incubation with lethal dose 10 (LD_10_) (white) and LD_50_ (grey) expressed as a percentage of the control (E=epirubicin, C=cisplatin, F=5-fluorouracil).

^3^H-OMG cellular uptake rate was reduced upon exposure to epirubicin, cisplatin and 5-FU, with the greatest reduction (35%) in ^3^H-OMG uptake resulting from 72 h exposure to LD_50_ epirubicin (Figure 3A and B). Forty-eight hours exposure to LD_50_ epirubicin, cisplatin and 5-FU resulted in a significant reduction in cellular ^3^H-OMG uptake compared to controls (*P*=0.037, *P*=0.005 and *P*=0.002 respectively) as did 72 h exposure (*P*=0.002, *P*=0.035 and *P*=0.001 respectively). LD_10_ 48 h exposure resulted in a non-significant decrease in ^3^H-OMG uptake for epirubicin, cisplatin and 5-FU (*P*=0.064, *P*=0.539 and *P*=0.05 respectively). LD_10_ exposure at 72 h caused a significant reduction in uptake for epirubicin (*P*=0.033) but not for cisplatin or 5-FU (*P*=0.304 and *P*=0.212 respectively).

### Effect of chemotherapy on cellular HK activity

**Figure 4:**
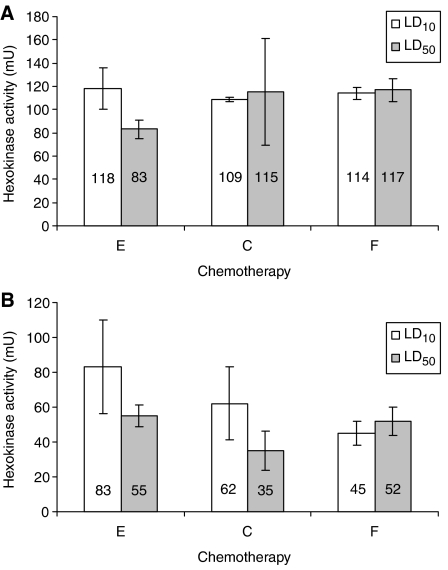
Cellular hexokinase (HK) activity, expressed as milliunits per milligram of cellular protein as a percentage of control (E=epirubicin, C=cisplatin, F=5-fluorouracil), after 48 (**A**) and 72 (**B**) h exposure to lethal dose 10 (LD_10_) (white) and LD_50_ (grey) doses of epirubicin, cisplatin and 5-FU (E=epirubicin, C=cisplatin, F=5-fluorouracil).

LD_10_ 48 h exposure to epirubicin, cisplatin and 5-FU resulted in a non-significant increase in cellular HK activity (Figure 4A and B) in comparison to controls (*P*=0.575, *P*=0.982 and *P*=0.465 respectively). Exposure to 48 h LD_50_, cisplatin and 5-FU also caused a non-significant increase in cellular HK activity (*P*=0.727 and *P*=0.282 respectively) whereas with epirubicin there was a non-significant reduction in HK activity (*P*=0.451).

Exposure to cisplatin and epirubicin for 72 h decreased HK activity, with the greatest reduction caused by exposure to LD_50_ cisplatin (65%). Hexokinase activity was significantly reduced by exposure for 72 h to cisplatin and 5-FU (cisplatin *P*=0.047 and *P*=0.011, 5-FU *P*=0.016 and *P*=0.018 respectively for LD_10_ and LD_50_). Exposure for 72 h to epirubicin caused a significant reduction in HK activity with the LD_50_ dose (*P*=0.02) but not the LD_10_ dose (*P*=0.202).

### Latent cytotoxicity of chemotherapeutic agents

**Figure 5:**
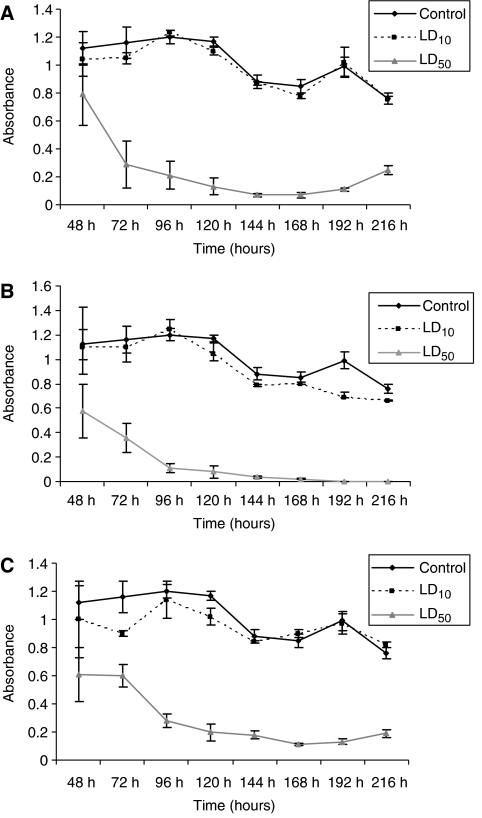
Determination of the growth inhibitory effect of each agent by performing MTT measurements 6 days after treatment with cisplatin (**A**), epirubicin (**B**) and 5-FU (**C**) for 72 h. Cell survival is expressed as absorbance as measured on spectrophotometer.

Following 72 h exposure to both LD_10_ and LD_50_ of each individual chemotherapeutic agent, cells were washed and fresh media was added followed by further incubation for up to 6 days. Cells were able to recover from exposure to 5-FU and cisplatin when the drug was removed. However, epirubicin-treated cells did not recover even after 6 days of incubation in fresh media (Figure 5A–C). Annexin V-PE flow cytometry revealed that AGS cells exposed to 72 h LD_50_ epirubicin resulted in 69% cell death, 39% of surviving cells were annexin V-PE positive and 7-AAD negative, indicating this group of cells were in the early stages of apoptosis (Table 3).

## DISCUSSION

Chemotherapy for tumours at or around the gastro-oesophageal junction is varied, with most regimes including one of epirubicin, cisplatin or 5-FU (Cunningham *et al*, 2006). All three chemotherapeutic agents caused a reduction in ^18^FDG uptake by AGS cells with epirubicin having the greatest effect, followed by 5-FU then cisplatin. Previous work on the AGS cell line using the same three chemotherapeutic agents and exposure times identified similar LD_50_ concentrations of each agent, 5-FU 10 *μ*g ml^−1^, cisplatin 10 *μ*g ml^−1^ and epirubicin 0.25 *μ*g ml^−1^ (Couper and Park, 2003). In common with previous studies, exposure to epirubicin and cisplatin induced dose-dependent G_1_ (LD_10_) and G_2_ (LD_50_) cell cycle arrest (Barry *et al*, 1990; Sorenson *et al*, 1990; Shapiro *et al*, 1998; Zoli *et al*, 2004). 5-FU exposure resulted in a build up of cells in S phase (LD_10_) and G_1_ (LD_50_). Epirubicin, an anthracycline derivative of doxorubicin, exerts its anti-tumour effects via its action as a DNA intercalating agent and as an inhibitor of topoisomerase II (Cersosimo and Hong, 1986; Bartkowiak *et al*, 1992; Zoli *et al*, 2004). The arrest of AGS cells at higher concentrations of epirubicin may be related to peak activity of topoisomerases occurring during the G_2_ phase (Chow and Ross, 1987). Exposure to cisplatin, an alkylating agent, results in the binding of cisplatin to DNA, forming cisplatin-DNA adducts which causes an alteration in the conformation of DNA leading to cell cycle arrest and apoptosis (Jordan and Carmo-Fonseca, 2000; Gonzalez *et al*, 2001; Wang *et al*, 2004). Cell cycle arrest following exposure to cisplatin occurs mainly within G_2_ (Eastman, 1990; Sorenson *et al*, 1990), although this may be tumour type dependent (Sekiguchi *et al*, 1996; Shapiro *et al*, 1998). The main mechanism of action of 5-FU is related to its conversion to 5-fluoro-2′-deoxy-5′-monophosphate (via the pyrimidine pathway) leading to the inhibition of thymidylate synthase and hence DNA synthesis (Pinedo and Peters, 1988). Studies have revealed an increase S-phase fraction in tumour cells, caused by 5-FU (Barry *et al*, 1990; Yamane *et al*, 1999; Park *et al*, 2004), including gastric carcinomas (Inada *et al*, 1997).

Dittmann *et al* (2002), evaluating ^18^FDG uptake in a squamous cell carcinoma oesophageal cell line, reported that 24 h incubation periods in concentrations of 5-FU and cisplatin, resulting in 22.8 and 60.6% cell death respectively, had no effect on cellular ^18^FDG uptake, following a 24 h period of incubation in chemotherapy-free media prior to uptake experiments. Furthermore, after 24 h exposure to these same concentrations of drugs the S-phase fraction was elevated considerably, yet had no impact on ^18^FDG uptake. Smith *et al* (2000) investigating tomudex, which is a more specific thymidylate synthase inhibitor than 5-FU, and oxaliplatin (a platinum agent similar to cisplatin) on a colonic tumour cell line found that exposure to tomudex for 24 and 48 h resulted in increasing levels of cellular ^3^H-DG uptake with increasing exposure to the agent. This increase in uptake was paralleled with an increase in the S-phase fraction. The contrasts between these studies and ours may be in part explained by Yamane *et al* (1999), who revealed that although increasingly lengthy exposure to 5-FU resulted in S-phase accumulation of colorectal cancer cells with increased apoptosis, the Ki-67 labelling index decreased. Therefore, this S-phase accumulation is not proliferative but lethal. Exposure to oxaliplatin (Smith *et al*, 2000) resulted in decreased levels of cellular ^3^H-DG uptake compared to controls with a varied cell cycle distribution. Although ^18^FDG uptake in this study was found to be decreased after treatment with each agent, the effect on cell cycle was agent-specific suggesting that the changes in ^18^FDG uptake are not cell cycle-specific, a finding previously reported by others (Higashi *et al*, 1993; Haberkorn *et al*, 1994).

Recently an analysis (Barros *et al*, 2005) based on the relationship between HK, glucose transport and intracellular glucose concentration shows that, at least in neuronal cells, the flux of glucose through the cell is dependent both on HK and glucose transport and that appreciable increases in flux require increases in both of these activities, whereas decreased flux can be brought about by a reduction in glucose transport or HK activity or both. Qualitatively, in our study, in each case where a decrease in ^18^FDG incorporation is observed there is a corresponding decrease in glucose transport. By far the greatest decrease in ^18^FDG incorporation is observed with the LD_50_ dose of epirubicin for 72 h, which also marginally shows the greatest reduction in glucose transport. This treatment also causes an appreciable decrease in HK activity, which may augment the effect of glucose transport on ^18^FDG incorporation. However, the lack of effect of the LD_10_ dose of 5-FU for 72 h on ^18^FDG incorporation or glucose transport, which caused a significant decrease in HK activity, suggests that glucose transport is the most important parameter for ^18^FDG incorporation in this cell line. Further, regression analysis performed on pooled 48 and 72 h data from each treatment shows a strong correlation between the changes in FDG incorporation and glucose transport (*t*=0.863, *n*=12, *P*<0.001) but not between changes in FDG incorporation and HK activity (*t*=0.31, *n*=12, *P*>0.1).

One possible reason for the closer association of glucose transport with ^18^FDG incorporation compared with HK activity may be that the glucose transport assay uses intact cells so is a true measure of the glucose transport of the AGS cell, whereas the HK assay is performed in cell homogenates. Since HK activity *in vivo* is compartmentalised and highly regulated (Smith, 2000), cell breakage is likely to disrupt these regulatory systems.

We found that ^18^FDG incorporation was consistently diminished by treatment with epirubicin, 5-FU or cisplatin. We did not see any evidence of increased incorporation. Although response to therapy is generally associated with decreased ^18^FDG incorporation, a number of studies (Basu and Alavi, 2007) have reported that ^18^FDG incorporation can increase in some responding tumours. In cases where this ‘metabolic flare’ phenomenon has been observed in breast tumours treated with anti-oestrogen treatment it has been attributed to the stimulatory effect of the anti-oestrogen at low blood concentrations (the situation when the patient begins therapy) on their cancer cells. A flare phenomenon occasionally found after radiotherapy generally corresponds with influx of inflammatory cells into the tumour (Kostakoglu and Goldsmith, 2004). Kubota *et al* (1992) studying ^18^FDG uptake in a malignant murine model discovered that ^18^FDG accumulated not only within the tumour cells, but also in the inflammatory components which appear with growth or tumour necrosis; however, the major source of ^18^FDG was still tumour cells. Clearly neither of these situations is relevant to our treatment/model type.

Another type of hypermetabolism associated with ^18^FDG has been reported in both solid tumours (Maruyama *et al*, 1999) and cell lines (Fujibayashi *et al*, 1997). This is likely to reflect biochemical changes within the cells in response to initial damage. We determined ^18^FDG incorporation at times corresponding to appreciable cell death so the initial response would have been complete.

Comparing ^18^FDG incorporation after 48 and 72 h of treatment, when expressed relative to cellular protein, the decrease in ^18^FDG incorporation is seen to plateau for treatment with cisplatin and 5-FU but not with epirubicin in which ^18^FDG incorporation continues to decline. This may be explained by the high proportion (77%) of apoptotic cells in cell populations treated with LD_50_ epirubicin for 72 h compared with cells exposed to cisplatin and 5-FU, suggesting that ^18^FDG incorporation by apoptotic cells is decreased compared with viable cells. Following on from this, determination of the growth inhibitory effect of each agent by performing MTT measurements 6 days after treatment with each agent showed that AGS cells can recover from treatment with 5-FU and cisplatin but not after treatment with epirubicin. In assessing two chemotherapy regimens for gastro-oesophageal cancer, Couper and Park (2003) noted that AGS cells exposed to a combination of LD_50_ of both cisplatin and 5-FU were able to recover following clearance of the chemotherapy. The addition of epirubicin to the combination of cisplatin and 5-FU resulted in a continual growth inhibitory effect (Couper and Park, 2003). The nature of Coupers' study meant that this effect may be due to the combined effect of three chemotherapeutic agents rather than specifically related to epirubicin. Engles *et al* treated MCF-7 breast carcinoma cells for 24 h with doxorubicin (an anthracycline similar to epirubicin) and 5-FU then re-incubated the cells in chemotherapy-free medium for a further 72 h. They found that cell number in populations treated with doxorubicin continued to decrease during the 72 h in doxorubicin-free medium (Engles *et al*, 2006), but addition of 5-FU-free media to MCF-7 cells treated for 24 h with 5-FU was associated with an increase in cell number indicative of recovery. It appears that the efficacy of epirubicin is associated with reduced cellular glycolytic rate (Zhou *et al*, 2002).

One of the limitations of our study is the extrapolation from *in vitro* to *in vivo*. Our studies are performed on well-perfused cells with a good nutrient and oxygen supply in an environment with neutral pH. Within solid tumours *in vivo* there are regions with compromised blood flow and consequent nutrient deprivation, lactic acid production and acidic pH. These are all factors that may influence ^18^FDG incorporation. Burgman *et al* (2001) showed that induction of hypoxia resulted in increased ^18^FDG incorporation by MCF-7 cells whilst HK activity is influenced by environmental pH (Miccoli *et al*, 1996). However, the region of tumour growth will have a good blood supply and is where most of the ^18^FDG is likely to reach.

To simulate the uptake of ^18^FDG within solid tumours, ^18^FDG uptake was also expressed as activity per flask. The uptake of ^18^FDG by detached cells could not be determined as steps to wash away non-incorporated ^18^FDG would involve centrifugation, which would be a problem with detached cells as these are generally late dying/dead cells with fragile or damaged cell membranes. However, cells that were undergoing early apoptosis, for example, cells treated with epirubicin, were still attached and would be included in the analyses. Furthermore, *in vivo*, dead cells are rapidly removed by macrophages. So the contribution of dead cells *in vivo* is likely to be small.

In summary, treatment of gastric adenocarcinoma cells with cisplatin, 5-FU and epirubicin results in decreased ^18^FDG incorporation. The greatest reduction in ^18^FDG uptake per cell is induced by epirubicin. In contrast to cisplatin and 5-FU treated cells, epirubicin-treated cells did not recover when the drug was removed from the medium, corresponding with the annexin V-PE results, suggesting that the level of change in ^18^FDG incorporation is predictive of tumour cell response. Each chemotherapeutic agent decreased glucose transport suggesting that glucose transport is the rate-limiting step for ^18^FDG incorporation by AGS cells.

## Figures and Tables

**Table 1:** Chemotherapy dose–time relationship and cell death

		**Cell death, % (±s.e.m.)**	
**Chemotherapy**	**Dose**	**48 h**	**72 h**
Epirubicin	LD_10_	4 (4)	5 (2)
	LD_50_	50 (1)	69 (10)
			
Cisplatin	LD_10_	5 (7)	5 (4)
	LD_50_	56 (7)	77 (12)
			
5-Fluorouracil	LD_10_	9 (8)	13 (9)
	LD_50_	43 (1)	55 (6)

Results of MTT assay performed at 48 and 72 h.

**Table 2:** Cell cycle analysis following exposure to chemotherapeutic agents

			**Cell cycle, % (±s.e.m.)**		
**Chemotherapy**	**Time (h)**	**Treatment**	**G_1_**	**S**	**G_2_**
Control	48		50 (1)	31 (1)	19 (1)
	72		35 (1)	30 (2)	27 (6)
					
5-Fluorouracil	48	LD_10_	36 (2)	48 (0)	14 (1)
		LD_50_	77 (1)	9 (0)	16 (1)
	72	LD_10_	14 (1)	64 (3)	19 (1)
		LD_50_	54 (2)	14 (3)	30 (2)
					
Cisplatin	48	LD_10_	57 (0)	24 (5)	19 (6)
		LD_50_	30 (1)	22 (0)	47 (1)
	72	LD_10_	48 (1)	27 (2)	24 (1)
		LD_50_	30 (0)	16 (0)	61 (0)
					
Epirubicin	48	LD_10_	59 (0)	21 (1)	21 (0)
		LD_50_	31 (1)	13 (3)	59 (2)
	72	LD_10_	46 (1)	25 (2)	28 (1)
		LD_50_	26 (2)	10 (1)	63 (2)

Flow cytometry was performed on a Becton Dickinson FACS Calibur (San Jose, CA, USA), using blue light (488 nm), detecting forward and 90° angle light scatter. Cell cycle analysis was performed using FlowJo v4.5.2 analysis software (Tree Star Inc., OR, USA), utilising the Dean–Jett–Fox model, analysing 10 000 events.

**Table 3:** Results of annexin V-PE flow cytometry upon 72 h exposure to chemotherapy

		**Cells, % (±s.e.m.)**		
**Chemotherapy**	**Dose**	**Annexin V-PE and 7-AAD negative**	**Annexin V-PE positive and 7-AAD negative**	**Annexin V-PE and 7-AAD positive**
Control		93 (1)	4 (1)	2 (0)
				
Epirubicin	LD_10_	94 (1)	2 (1)	4 (0)
	LD_50_	23 (1)	39 (1)	38 (0)
				
Cisplatin	LD_10_	95 (0)	3 (0)	2 (0)
	LD_50_	80 (2)	15 (2)	6 (0)
				
5-Fluorouracil	LD_10_	89 (0)	7 (0)	4 (0)
	LD_50_	93 (2)	3 (1)	4 (2)

Annexin V-PE FACS, 10 000 events counted per sample with results displayed as a percentage of valid counts (annexin V-PE and 7-AAD negative=healthy cells; annexin V-PE positive and 7-AAD negative=early apoptosis; annexin V-PE and 7-AAD positive=necrosis or late apoptosis).

## References

[bib1] Al-Saeedi F, Welch AE, Smith TA (2005) [methyl-(3)H]Choline incorporation into MCF7 tumour cells: correlation with proliferation. Eur J Nucl Med Mol Imaging 32: 660–667 15660258 10.1007/s00259-004-1707-6

[bib2] Aloj L, Caraco C, Jagoda E, Eckelman WC, Neumann RD (1999) Glut-1 and hexokinase expression: relationship with 2-fluoro-2-deoxy-D-glucose uptake in A431 and T47D cells in culture. Cancer Res 59: 4709–4714 10493529

[bib3] Barros LF, Porras OH, Bittner CX (2005) Why glucose transport in the brain matters for PET. Trends Neurosci 28: 117–119 15749163 10.1016/j.tins.2005.01.002

[bib4] Barry MA, Behnke CA, Eastman A (1990) Activation of programmed cell death (apoptosis) by cisplatin, other anticancer drugs, toxins and hyperthermia. Biochem Pharmacol 40: 2353–2362 2244936 10.1016/0006-2952(90)90733-2

[bib5] Bartkowiak D, Hemmer J, Rottinger E (1992) Dose dependence of the cytokinetic and cytotoxic effects of epirubicin *in vitro*. Cancer Chemother Pharmacol 30: 189–192 1628367 10.1007/BF00686310

[bib6] Basu S, Alavi A (2007) Defining co-related parameters between ‘metabolic’ flare and ‘clinical’, ‘biochemical’, and ‘osteoblastic’ flare and establishing guidelines for assessing response to treatment in cancer. Eur J Nucl Med Mol Imaging 34: 441–443 17072613 10.1007/s00259-006-0264-6

[bib7] Brown RS, Leung JY, Kison PV, Zasadny KR, Flint A, Wahl RL (1999) Glucose transporters and FDG uptake in untreated primary human non-small cell lung cancer. J Nucl Med 40: 556–565 10210213

[bib8] Burgman P, Odonoghue JA, Humm JL, Ling CC (2001) Hypoxia-Induced increase in FDG uptake in MCF7 cells. J Nucl Med 42: 170–175 11197971

[bib9] Burrows RC, Freeman SD, Charlop AW, Wiseman RW, Adamsen TC, Krohn KA, Spence AM (2004) [18F]-2-fluoro-2-deoxyglucose transport kinetics as a function of extracellular glucose concentration in malignant glioma, fibroblast and macrophage cells *in vitro*. Nucl Med Biol 31: 1–9 14741565 10.1016/s0969-8051(02)00449-3

[bib10] Carmichael J, DeGraff WG, Gazdar AF, Minna JD, Mitchell JB (1987) Evaluation of a tetrazolium-based semiautomated colorimetric assay: assessment of chemosensitivity testing. Cancer Res 47: 936–942 3802100

[bib11] Cersosimo RJ, Hong WK (1986) Epirubicin: a review of the pharmacology, clinical activity, and adverse effects of an adriamycin analogue. J Clin Oncol 4: 425–439 3005521 10.1200/JCO.1986.4.3.425

[bib12] Chow KC, Ross WE (1987) Topoisomerase-specific drug sensitivity in relation to cell cycle progression. Mol Cell Biol 7: 3119–3123 2823120 10.1128/mcb.7.9.3119-3123.1987 PMC367945

[bib13] Cloherty EK, Levine KB, Graybill C, Carruthers A (2002) Cooperative nucleotide binding to the human erythrocyte sugar transporter. Biochemistry 41: 12639–12651 12379106 10.1021/bi0259002

[bib14] Couper G, Park K (2003) *In vitro* response to chemotherapy: evidence of resistance to neo-adjuvant chemotherapy in upper gastrointestinal cancer. Br J Surg 90: 50

[bib15] Couper GW, McAteer D, Wallis F, Norton M, Welch A, Nicolson M, Park KG (1998) Detection of response to chemotherapy using positron emission tomography in patients with oesophageal and gastric cancer. Br J Surg 85: 1403–1406 9782025 10.1046/j.1365-2168.1998.00963.x

[bib16] Cunningham D, Allum WH, Stenning SP, Thompson JN, Van de Velde CJ, Nicolson M, Scarffe JH, Lofts FJ, Falk SJ, Iveson TJ, Smith DB, Langley RE, Verma M, Weeden S, Chua YJ, Participants MT (2006) Perioperative chemotherapy *vs* surgery alone for resectable gastroesophageal cancer. N Engl J Med 355: 11–20 16822992 10.1056/NEJMoa055531

[bib17] Dittmann H, Dohmen BM, Kehlbach R, Bartusek G, Pritzkow M, Sarbia M, Bares R (2002) Early changes in [18F]FLT uptake after chemotherapy: an experimental study. Eur J Nucl Med Mol Imaging 29: 1462–1469 12397465 10.1007/s00259-002-0925-z

[bib18] Eastman A (1990) Activation of programmed cell death by anticancer agents: cisplatin as a model system. Cancer Cells 2: 275–280 2223389

[bib19] Engles JM, Quarless SA, Mambo E, Ishimori T, Cho SY, Wahl RL (2006) Stunning and its effect on 3H-FDG uptake and key gene expression in breast cancer cells undergoing chemotherapy. J Nucl Med 47: 603–608 16595493

[bib20] Fujibayashi Y, Waki A, Sakahara H, Konishi J, Yonekura Y, Ishii Y, Yokoyama A (1997) Transient increase in glycolytic metabolism in cultured tumor cells immediately after exposure to ionizing radiation: from gene expression to deoxyglucose uptake. Radiat Res 147: 729–734 9189172

[bib21] Gallagher BM, Fowler JS, Gutterson NI, MacGregor RR, Wan CN, Wolf AP (1978) Metabolic trapping as a principle of oradiopharmaceutical design: some factors resposible for the biodistribution of [18F] 2-deoxy-2-fluoro-D-glucose. J Nucl Med 19: 1154–1161 214528

[bib22] Gilbert FJ, Park K, Thompson AM (2002) Scottish Audit of Gastric and Oesophageal Cancer: Report 1997–2000 – A prospective audit. http://www.show.scot.nhs.uk/crag/

[bib23] Gonzalez VM, Fuertes MA, Alonso C, Perez JM (2001) Is cisplatin-induced cell death always produced by apoptosis? Mol Pharmacol 59: 657–663 11259608 10.1124/mol.59.4.657

[bib24] Haberkorn U, Oberdorfer F, Klenner T, Strauss LG, Stohr M, Wallich R, Altmann A, Kaick GV (1994) Metabolic and transcriptional changes in osteosarcoma cells treated with chemotherapeutic drugs. Nucl Med Biol 21: 835–845 9234333 10.1016/0969-8051(94)90163-5

[bib25] Higashi K, Clavo AC, Wahl RL (1993) Does FDG uptake measure proliferative activity of human cancer cells? *In vitro* comparison with DNA flow cytometry and tritiated thymidine uptake. J Nucl Med 34: 414–419 8441032

[bib26] Higashi T, Tamaki N, Honda T, Torizuka T, Kimura T, Inokuma T, Ohshio G, Hosotani R, Imamura M, Konishi J (1997) Expression of glucose transporters in human pancreatic tumors compared with increased FDG accumulation in PET study. J Nucl Med 38: 1337–1344 9293783

[bib27] Inada T, Ichikawa A, Igarashi S, Kubota T, Ogata Y (1997) Effect of preoperative 5-fluorouracil on apoptosis of advanced gastric cancer. J Surg Oncol 65: 106–110 9209521 10.1002/(sici)1096-9098(199706)65:2<106::aid-jso6>3.0.co;2-b

[bib28] Jordan P, Carmo-Fonseca M (2000) Molecular mechanisms involved in cisplatin cytotoxicity. Cell Mol Life Sci 57: 1229–1235 11028915 10.1007/PL00000762 PMC11147023

[bib29] Kostakoglu L, Goldsmith SJ (2004) PET in the assessment of therapy response in patients with carcinoma of the head and neck and of the esophagus. J Nucl Med 45: 56–68 14734674

[bib30] Kroep JR, Van Groeningen CJ, Cuesta MA, Craanen ME, Hoekstra OS, Comans EF, Bloemena E, Hoekstra CJ, Golding RP, Twisk JW, Peters GJ, Pinedo HM, Lammertsma AA (2003) Positron emission tomography using 2-deoxy-2-[18F]-fluoro-D-glucose for response monitoring in locally advanced gastroesophageal cancer; a comparison of different analytical methods. Mol Imaging Biol 5: 337–346 14630513 10.1016/j.mibio.2003.09.007

[bib31] Kubota R, Yamada S, Kubota K, Ishiwata K, Tamahashi N, Ido T (1992) Intratumoral distribution of fluorine-18-fluorodeoxyglucose *in vivo*: high accumulation in macrophages and granulation tissues studied by microautoradiography. J Nucl Med 33: 1972–1980 1432158

[bib32] Kurokawa T, Yoshida Y, Kawahara K, Tsuchida T, Okazawa H, Fujibayashi Y, Yonekura Y, Kotsuji F (2004) Expression of GLUT-1 glucose transfer, cellular proliferation activity and grade of tumor correlate with [F-18]-fluorodeoxyglucose uptake by positron emission tomography in epithelial tumors of the ovary. Int J Cancer 109: 926–932 15027127 10.1002/ijc.20057

[bib33] Maruyama I, Sadato N, Waki A, Tsuchida T, Yoshida M, Fujibayashi Y, Ishii Y, Kubota T, Yonekura Y (1999) Hyperacute changes in glucose metabolism of brain tumors after stereotactic radiosurgery: a PET study. J Nucl Med 40: 1085–1090 10405124

[bib34] Mathupala SP, Rempel A, Pedersen PL (1997) Aberrant glycolytic metabolism of cancer cells: a remarkable coordination of genetic, transcriptional, post-translational, and mutational events that lead to a critical role for type II hexokinase. J Bioenerg Biomembr 29: 339–343 9387094 10.1023/a:1022494613613

[bib35] Miccoli L, Oudard S, Sureau F, Poirson F, Dutrillaux B, Poupon MF (1996) Intracellular pH governs the subcellular distribution of hexokinase in a glioma cell line. Biochem J 313(Part 3): 957–962 8611181 10.1042/bj3130957 PMC1217004

[bib36] Mosmann T (1983) Rapid colorimetric assay for cellular growth and survival: application to proliferation and cytotoxicity assays. J Immunol Methods 65: 55–63 6606682 10.1016/0022-1759(83)90303-4

[bib37] Mueckler M (1994) Facilitative glucose transporters. Eur J Biochem 219: 713–725 8112322 10.1111/j.1432-1033.1994.tb18550.x

[bib38] Nelson CA, Wang JQ, Leav I, Crane PD (1996) The interaction among glucose transport, hexokinase, and glucose-6-phosphatase with respect to 3H-2-deoxyglucose retention in murine tumor models. Nucl Med Biol 23: 533–541 8832712 10.1016/0969-8051(96)00037-6

[bib39] Ott K, Fink U, Becker K, Stahl A, Dittler HJ, Busch R, Stein H, Lordick F, Link T, Schwaiger M, Siewert JR, Weber WA (2003) Prediction of response to preoperative chemotherapy in gastric carcinoma by metabolic imaging: results of a prospective trial. J Clin Oncol 21: 4604–4610 14673049 10.1200/JCO.2003.06.574

[bib40] Park JK, Lee SH, Kang JH, Nishio K, Saijo N, Kuh HJ (2004) Synergistic interaction between gefitinib (Iressa, ZD1839) and paclitaxel against human gastric carcinoma cells. Anticancer Drugs 15: 809–818 15494644 10.1097/00001813-200409000-00011

[bib41] Pinedo HM, Peters GF (1988) Fluorouracil: biochemistry and pharmacology. J Clin Oncol 6: 1653–1664 3049954 10.1200/JCO.1988.6.10.1653

[bib42] Sekiguchi I, Suzuki M, Tamada T, Shinomiya N, Tsuru S, Murata M (1996) Effects of cisplatin on cell cycle kinetics, morphological change, and cleavage pattern of DNA in two human ovarian carcinoma cell lines. Oncology 53: 19–26 8570126 10.1159/000227529

[bib43] Shapiro GI, Edwards CD, Ewen ME, Rollins BJ (1998) p16INK4A participates in a G1 arrest checkpoint in response to DNA damage. Mol Cell Biol 18: 378–387 9418885 10.1128/mcb.18.1.378 PMC121508

[bib44] Smith TA (1999) Facilitative glucose transporter expression in human cancer tissue. Br J Biomed Sci 56: 285–292 10795374

[bib45] Smith TA (2000) Mammalian hexokinases and their abnormal expression in cancer. Br J Biomed Sci 57: 170–178 10912295

[bib46] Smith TA, Maisey NR, Titley JC, Jackson LE, Leach MO, Ronen SM (2000) Treatment of SW620 cells with Tomudex and oxaliplatin induces changes in 2-deoxy-D-glucose incorporation associated with modifications in glucose transport. J Nucl Med 41: 1753–1759 11038008

[bib47] Smith TA, Sharma RI, Thompson AM, Paulin FE (2006) Tumor 18F-FDG incorporation is enhanced by attenuation of P53 function in breast cancer cells *in vitro*. J Nucl Med 47: 1525–1530 16954562

[bib48] Sorenson CM, Barry MA, Eastman A (1990) Analysis of events associated with cell cycle arrest at G2 phase and cell death induced by cisplatin. J Natl Cancer Inst 82: 749–755 1691303 10.1093/jnci/82.9.749

[bib49] Tohma T, Okazumi S, Makino H, Cho A, Mochiduki R, Shuto K, Kudo H, Matsubara K, Gunji H, Ochiai T (2005) Relationship between glucose transporter, hexokinase and FDG-PET in esophageal cancer. Hepatogastroenterology 52: 486–490 15816463

[bib50] Torizuka T, Tamaki N, Inokuma T, Magata Y, Sasayama S, Yonekura Y, Tanaka A, Yamaoka Y, Yamamoto K, Konishi J (1995) *In vivo* assessment of glucose metabolism in hepatocellular carcinoma with FDG-PET. J Nucl Med 36: 1811–1817 7562048

[bib51] Waki A, Kato H, Yano R, Sadato N, Yokoyama A, Ishii Y, Yonekura Y, Fujibayashi Y (1998) The importance of glucose transport activity as the rate-limiting step of 2-deoxyglucose uptake in tumor cells *in vitro*. Nucl Med Biol 25: 593–597 9804039 10.1016/s0969-8051(98)00038-9

[bib52] Wang G, Reed E, Li QQ (2004) Molecular basis of cellular response to cisplatin chemotherapy in non-small cell lung cancer (Review). Oncol Rep 12: 955–965 15492778

[bib53] Warburg O (1956) On the origin of cancer cells. Science 123: 306–314 10.1126/science.123.3191.309 13298683

[bib54] Weber G, Cantero A (1955) Glucose-6-phosphatase activity in normal, pre-cancerous, and neoplastic tissues. Cancer Res 15: 105–108 14352196

[bib55] Weber WA, Ott K, Becker K, Dittler HJ, Helmberger H, Avril NE, Meisetschlager G, Busch R, Siewert JR, Schwaiger M, Fink U (2001) Prediction of response to preoperative chemotherapy in adenocarcinomas of the esophagogastric junction by metabolic imaging. J Clin Oncol 19: 3058–3065 11408502 10.1200/JCO.2001.19.12.3058

[bib56] Yamane N, Makino M, Kaibara N (1999) S-phase accumulation precedes apoptosis induced by preoperative treatment with 5-fluorouracil in human colorectal carcinoma cells. Cancer 85: 309–317 10023697 10.1002/(sici)1097-0142(19990115)85:2<309::aid-cncr7>3.0.co;2-x

[bib57] Younes M, Lechago LV, Somoano JR, Mosharaf M, Lechago J (1996) Wide expression of the human erythrocyte glucose transporter Glut1 in human cancers. Cancer Res 56: 1164–1167 8640778

[bib58] Zhao S, Kuge Y, Mochizuki T, Takahashi T, Nakada K, Sato M, Takei T, Tamaki N (2005) Biologic correlates of intratumoral heterogeneity in 18F-FDG distribution with regional expression of glucose transporters and hexokinase-II in experimental tumor. J Nucl Med 46: 675–682 15809491

[bib59] Zhao S, Kuge Y, Tsukamoto E, Mochizuki T, Kato T, Hikosaka K, Nakada K, Hosokawa M, Kohanawa M, Tamaki N (2002) Fluorodeoxyglucose uptake and glucose transporter expression in experimental inflammatory lesions and malignant tumours: effects of insulin and glucose loading. Nucl Med Commun 23: 545–550 12029209 10.1097/00006231-200206000-00006

[bib60] Zhou R, Vander Heiden MG, Rudin CM (2002) Genotoxic exposure is associated with alterations in glucose uptake and metabolism. Cancer Res 62: 3515–3520 12067998

[bib61] Zoli W, Ricotti L, Tesei A, Ulivi P, Gasperi Campani A, Fabbri F, Gunelli R, Frassineti GL, Amadori D (2004) Schedule-dependent cytotoxic interaction between epidoxorubicin and gemcitabine in human bladder cancer cells *in vitro*. Clin Cancer Res 10: 1500–1507 14977854 10.1158/1078-0432.ccr-1107-03

